# Contrast-enhanced ultrasound versus CT angiography for endoleak detection after EVAR: a reference-standard-aware systematic review and meta-analysis

**DOI:** 10.1186/s42155-026-00731-6

**Published:** 2026-07-22

**Authors:** Rafaella Brandão de Melo Soares, Rafael de Negreiros Botan, Iruena Moraes Kessler

**Affiliations:** 1 Postgraduate Program in Medical Sciences, Faculty of Medicine, University of Brasilia, Brasilia, DF 70910-900 Brazil; 2 Faculty of Medicine, University of Brasilia, Brasilia, DF 70910-900 Brazil; 3 Neurosurgery Service, University Hospital of Brasilia, Brasilia, DF 70910-900 Brazil

**Keywords:** Abdominal aortic aneurysm, Endovascular aneurysm repair, Endoleak, Contrast-enhanced ultrasound, CT angiography, Diagnostic accuracy, Systematic review

## Abstract

**Purpose:**

To evaluate contrast-enhanced ultrasound (CEUS) for endoleak detection after endovascular aneurysm repair (EVAR) against CT angiography (CTA) or CTA-centered composite references, explicitly treating CTA as an imperfect reference standard.

**Methods:**

We searched PubMed/MEDLINE, Europe PMC, Crossref, OpenAlex, and performed an institutional Embase/Scopus coverage audit through 13 May 2026, with citation checking from prior reviews and included-study bibliographies. Eligible studies included adults undergoing paired CEUS and CTA or CTA-centered reference imaging after EVAR. The primary estimand was apparent CEUS diagnostic performance for any endoleak at the paired-examination level. An approximate bivariate logit-normal random-effects model was used as the primary meta-analysis; separate logit random-effects models, leave-one-out analysis, source-basis sensitivity analysis, and paired discordance testing were prespecified support analyses.

**Results:**

Eighteen all-endoleak studies contributed 2064 participants and 2452 paired examinations. The bivariate model estimated CEUS sensitivity as 0.905 (95% CI 0.869–0.932) and specificity as 0.943 (95% CI 0.890–0.971). Separate logit random-effects estimates were similar: sensitivity 0.909 (95% CI 0.873–0.936; *I*^2^ = 42.5%) and specificity 0.944 (95% CI 0.892–0.972; *I*^2^ = 88.7%). Results were consistent in ≤ 30-day and CTA-only subsets. CEUS-positive/reference-negative discordance exceeded CEUS-negative/reference-positive discordance (116 vs 67; ratio 1.73; *p* = 0.00036).

**Conclusions:**

CEUS shows high apparent diagnostic performance for post-EVAR endoleak detection against CTA-centered references, with sensitivity and specificity both approximately 91–94%. CEUS-positive/reference-negative discordance was more frequent than the reverse and may represent low-flow or delayed endoleaks not captured by CTA-centered reference imaging in some cases. However, substantial specificity heterogeneity and CTA-reference limitations argue against unqualified CTA replacement. CEUS is best supported as a CTA-sparing surveillance and adjudication tool in selected pathways, with CTA retained for baseline structural assessment, graft evaluation, procedural planning, and discordant or high-risk findings.

**Graphical Abstract:**

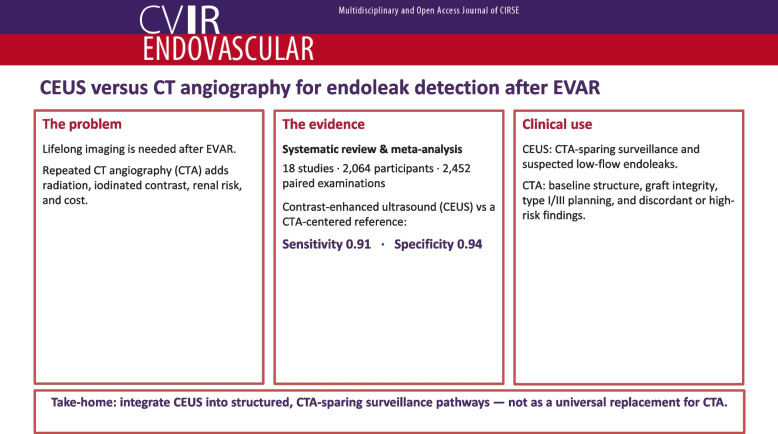

**Supplementary Information:**

The online version contains supplementary material available at 10.1186/s42155-026-00731-6.

## Background

Endovascular aneurysm repair (EVAR) has become a central treatment option for abdominal aortic aneurysm, but the durability of EVAR depends on lifelong surveillance. Endoleak is the most frequent EVAR-specific complication and may lead to aneurysm sac enlargement, reintervention, and rarely rupture. Current guidelines continue to place CTA, duplex ultrasound, and CEUS within post-EVAR follow-up pathways, with CT often emphasized early after repair and ultrasound-based surveillance used in longer-term follow-up when findings are stable or when contrast/radiation exposure is a concern [1–3].

CTA has major advantages: wide availability, reproducible anatomic mapping, evaluation of graft position and integrity, and procedural planning. It also has material disadvantages in a population that may require repeated imaging over the years: cumulative radiation, iodinated contrast exposure, renal risk, cost, and occasional beam-hardening or timing artifacts. CEUS addresses several of these limitations. It uses intravascular microbubble contrast, provides real-time dynamic flow assessment, avoids ionizing radiation, and can detect delayed or low-flow enhancement that may be missed on a static CT acquisition.

The difficulty is methodological. Many CEUS studies report diagnostic accuracy using CTA as the “gold standard”, yet CTA itself is imperfect for slow-flow type II endoleaks, post-embolization artifacts, timing-dependent enhancement, and endoleak classification. Conversely, CEUS is operator-dependent and can be limited by bowel gas, obesity, acoustic shadowing, and incomplete structural assessment of the endograft. A review that simply pools CEUS sensitivity and specificity against CTA risks overstating certainty in both directions: it may classify some CEUS-positive/reference-negative findings as false positives when CEUS is correct, while also failing to distinguish clinically urgent type I/III endoleaks from lower-risk type II endoleaks.

Prior reviews reported high CEUS performance, but did not foreground CTA-reference imperfection, repeated-examination clustering, or discordance directionality [4, 5]. Conventional pooling can obscure the methodological problem created when CTA is treated as an unquestioned reference. The present review adds a lock-date search through 13 May 2026, an institutional Embase/Scopus audit, explicit handling of clustered paired examinations, and a paired discordance analysis to characterize the asymmetry between CEUS-positive/reference-negative and CEUS-negative/reference-positive findings. It uses a reference-standard-aware framework: CTA is treated as the conventional comparator and a CTA-centered reference, not as an unquestioned truth standard. The objective was to estimate the apparent diagnostic performance and paired discordance profile of CEUS for post-EVAR endoleak detection and, on that basis, to evaluate the value of CEUS—as a complementary or potentially stand-alone test—during post-EVAR surveillance, clarifying its clinically defensible role within structured follow-up pathways.

## Methods

### Design and reporting framework

This systematic review was designed as a diagnostic test accuracy review following PRISMA-DTA and PRISMA-S principles [6, 7]. QUADAS-2 was used as the main risk-of-bias framework [8], with QUADAS-C concepts applied to comparative diagnostic accuracy issues where CEUS and CTA were evaluated in the same clinical pathway [9].

The review question was: among adults undergoing imaging surveillance after EVAR for abdominal or aortoiliac aneurysm, what is the apparent diagnostic performance and paired discordance pattern of CEUS compared with CTA or a CTA-centered reference for endoleak detection?

### Eligibility criteria

Studies were eligible for the primary quantitative synthesis if they

- Included adults after EVAR or aortoiliac EVAR surveillance.
- Compared CEUS with CTA or a CTA-centered composite/adjudicated reference.
- Reported, or allowed reconstruction of, a 2 × 2 table for any endoleak.
- Used paired or near-paired imaging.
- Evaluated postoperative surveillance rather than intraoperative imaging alone.

Studies were excluded from the primary quantitative synthesis, but considered for narrative or secondary discussion, if they were restricted to a single endoleak subtype, used experimental 3D-only or SMI-centered protocols without a clean all-endoleak CEUS-vs-CTA 2 × 2 table, did not provide reconstructible 2 × 2 data, used a highly selected adjudication framework that was not interchangeable with CTA-reference studies, or were case reports, educational reviews, technical notes, comments, or economic models without primary paired diagnostic data.

The Curti et al. study [10] was prespecified as secondary because type I and type III endoleaks were treated and excluded before the ultrasound comparison, leaving a type-II-only dataset. Morell-Hofert et al. [11], Jagdeesh et al. [12], Benedetto et al. [13], Frenzel et al. [14], Panagrosso et al. [15], and Snyder et al. [16] were prespecified as narrative contemporary evidence when they did not provide a simple all-endoleak CEUS-vs-CTA 2 × 2 table suitable for the primary pool, used adjudication/DSA or selected cohorts in a way that changed the estimand, or focused on treatment/intraoperative use rather than routine surveillance.

### Search strategy and study selection

Searches were run on 12 May 2026 and re-run on 13 May 2026 as a lock-date public search using PubMed/MEDLINE, Europe PMC, Crossref, and OpenAlex. Embase and Scopus were additionally audited through CAPES/UNB institutional access. Web of Science could not be exported at the lock date because access/export verification was not completed; this was mitigated by citation checking of prior reviews, included studies, and contemporary full texts where available. Search strings combined EVAR/aortic aneurysm terms, endoleak terms, CEUS/contrast-enhanced ultrasound terms, and CTA/CT angiography terms. Search metadata and platform-specific limitations are provided in Supplementary Table S1.

The public lock-date search identified 986 raw records: PubMed 376, Europe PMC 310, Crossref 100, and OpenAlex 200. The institutional audit identified 1222 Embase records and 142 Scopus records. After deduplication across public and institutional sources, 1654 unique records remained. Automated and manual title/abstract prioritization retained 185 potentially relevant records for relevance assessment. The final synthesis includes 18 all-endoleak quantitative studies, one type-II-only secondary study, and 15 narrative/context studies. The institutional audit changed the quantitative pool by adding one abstract-level poolable study from Embase and, more importantly, prompted re-adjudication of older public/citation-check records that had previously been underused.

The review was not prospectively registered because it originated from a completed master’s dissertation: the literature search and data extraction had already begun when the present article was designed, so the project did not meet the requirement for registration before the start of data extraction. Before final model execution, a structured analysis plan, extraction table, risk-of-bias matrix, and reproducible analysis scripts were frozen in the supplementary package. The full data and code are shared to allow independent reproduction. Screening and extraction used a structured, reproducible workflow rather than duplicate independent screening of every record. Eligibility decisions and all 2 × 2 tables for included quantitative studies were subsequently rechecked against the available source report, abstract, or documented reconstruction basis before final analysis; uncertain reconstructions were resolved by consensus.

### Data extraction

For each eligible study, we extracted first author, year, country, design, number of participants, number of paired examinations, CEUS-CTA interval, reference standard, scope of endoleak assessment, and 2 × 2 counts. Where studies reported sensitivity, specificity, PPV, NPV, and marginal counts, 2 × 2 values were reconstructed arithmetically.

The primary 2 × 2 convention was

- True positive: CEUS positive and reference positive.
- False negative: CEUS negative and reference positive.
- False positive: CEUS positive and reference negative.
- True negative: CEUS negative and reference negative.

The terms false positive and false negative are used here only within the reference-standard convention. They should not be read as proof that CTA or CEUS was clinically correct in discordant cases.

### Risk of bias and applicability

Risk of bias was assessed for all 18 primary quantitative studies using QUADAS-2, with comparative applicability issues mapped using QUADAS-C principles. Recurrent concerns included unclear blinding between index and reference tests, CTA as an imperfect reference, retrospective selection in several studies, intervals longer than the ideal paired window in Park et al., and repeated paired examinations per patient in several cohorts. Applicability concerns were greatest for older technology, small cohorts, subtype-restricted studies, and highly selected contemporary cohorts with sac expansion or post-intervention artifact.

### Statistical analysis

The primary DTA synthesis used an approximate bivariate logit-normal random-effects model implemented in Python 3.13 with SciPy 1.17.1. Study-level TP, FN, FP, and TN counts were converted to logit sensitivity and logit specificity with within-study binomial variances; a 0.5 continuity correction was applied when any 2 × 2 cell was zero. The model jointly summarized sensitivity and specificity while allowing between-study heterogeneity in both parameters. This implementation should be interpreted as an approximate bivariate logit-normal model, not a full binomial generalized linear mixed model. Separate univariate logit random-effects models were retained as support analyses because several studies contained zero cells and because patient-level clustered data were unavailable.

Heterogeneity was summarized with *I*^2^ for the univariate support models. Prespecified sensitivity analyses were studies with reported CEUS-reference interval ≤ 30 days, CTA-only reference studies, low repetition risk studies defined as paired examinations/participants ≤ 1.10, and source-basis sensitivity excluding abstract-level reconstructed studies. Leave-one-out analyses examined influential studies, especially for specificity heterogeneity. A Deeks-type funnel asymmetry regression was performed as an exploratory small-study-effect assessment because *k* = 18 [17]. A type-II-only secondary analysis was retained for Curti et al. but not pooled with the all-endoleak primary endpoint.

Paired discordance was summarized as CEUS-positive/reference-negative versus CEUS-negative/reference-positive counts, with an exact binomial test for imbalance among discordant pairs. This analysis is descriptive because the reference standard is imperfect.

## Results

### Search and included evidence

The combined lock-date deduplicated search set contained 1654 unique records, of which 185 were retained after structured title/abstract prioritization. Eighteen all-endoleak studies were eligible for the primary quantitative synthesis: Motta 2012, Perini 2011, Johnsen 2020, Park 2022, Gurtler 2013, Ten Bosch 2010, Houdek 2015, Bredahl 2016, Faccioli 2018, Clevert 2008, Henao 2006, Iezzi 2009, David 2016, Cantisani 2011, Cai 2017, Ma 2023, Wang 2024, and Nijhawan 2024 [18–35]. Table 1 summarizes the study-level audit matrix and the main reference-standard concerns for the prespecified quantitative subsets.

**Table 1:** Summary audit matrix for quantitative studies

Subset	Studies	Participants	Paired exams	Key concern
Broad all-endoleak primary pool	18	2064	2452	CTA-centered reference; repeated exams in several studies
Reported interval ≤ 30 days	12	1758	2026	Still includes repeated exams; specificity heterogeneity persists
CTA-only reference	16	1938	2319	CTA imperfection remains; excludes Wang and Nijhawan
Low repetition risk	11	1484	1484	Fewer studies; discordance imbalance attenuates
Type-II-only secondary evidence	1	119	119	Not comparable to all-endoleak endpoint

These studies contributed 2064 participants and 2452 paired examinations. The number of paired examinations per study ranged from 20 to 395. Twelve studies had reported CEUS-reference intervals ≤ 30 days and contributed 1758 participants and 2026 paired examinations. The reference model for the primary estimand was CTA-only in 16 studies and CTA-centered composite/adjudicated in 2 studies. One type-II-only study and 15 narrative/context studies were retained outside the primary pool because they changed the estimand, lacked a compatible 2 × 2 table, overlapped with an included report, or focused on selected/interventional/intraoperative scenarios.

### Study characteristics and audit matrix

Most studies were prospective or retrospective cohort studies from Europe, Asia, Brazil, and the United States. The unit of analysis was frequently paired examinations rather than unique patients. Seven primary studies (Motta 2012, Johnsen 2020, Gurtler 2013, Ten Bosch 2010, Houdek 2015, Ma 2023, and Nijhawan 2024) had moderate or high clustering risk because the number of paired examinations exceeded participants by more than 10% or because repeated follow-up visits were included; these studies contributed 968 of the 2452 paired examinations (39.5%). Because patient-level data were unavailable, repeated examinations within these cohorts were treated as independent, which is expected to narrow within-study confidence intervals and inflate the apparent precision of the pooled estimates. The prespecified low-repetition-risk analysis (11 studies with a paired-examination-to-participant ratio of 1.10 or less; 1484 examinations) was designed to address this concern (see “Sensitivity analyses” section).

### Risk of bias and applicability

QUADAS-2 assessment was completed for all 18 primary quantitative studies. Patient selection was low risk in 3 studies, unclear in 13, and high risk in 2. Index-test risk was unclear in all 18 because blinding and interpretation thresholds were incompletely reported in the available source basis. Reference-standard concerns were rated as unclear in all 18 because most studies used CTA as the conventional comparator without uniform multiphasic protocols, longitudinal adjudication, DSA confirmation, or sac-behavior confirmation for discordant cases. Flow-and-timing risk was low in 12 studies, unclear in 4, and high in 2. Applicability concerns were low in 10 studies and moderate in 8, mainly because of older CEUS technology, subtype or selected-cohort issues, and repeated paired examinations.

### Primary diagnostic performance

Across the 18 all-endoleak studies, CEUS identified 697 of 764 reference-positive paired observations and 1572 of 1688 reference-negative paired observations. The raw sensitivity was 0.912 and raw specificity was 0.931.

**Fig. 1:**
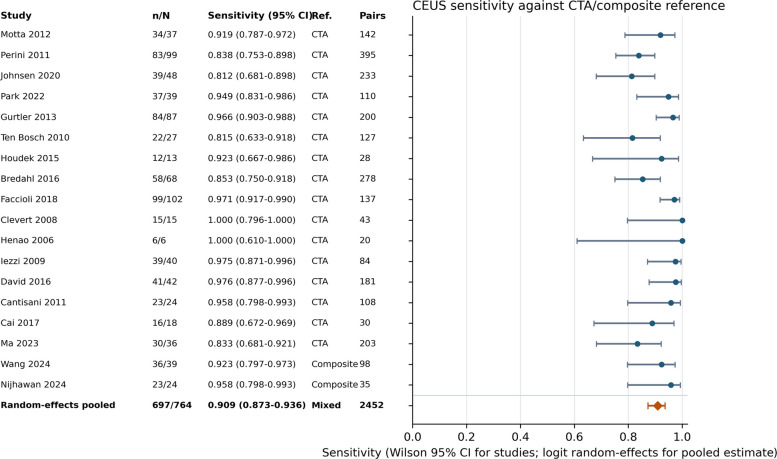
Forest plot of CEUS sensitivity for any endoleak. Study-specific sensitivity estimates are shown with 95% confidence intervals for the 18 all-endoleak studies

**Fig. 2:**
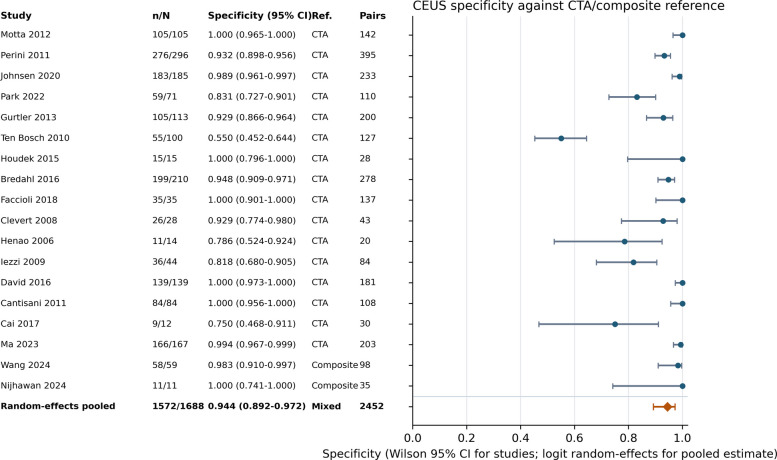
Forest plot of CEUS specificity for any endoleak. Study-specific specificity estimates are shown with 95% confidence intervals for the same 18 all-endoleak studies

Bivariate random-effects modeling estimated sensitivity as 0.905 (95% CI 0.869–0.932) and specificity as 0.943 (95% CI 0.890–0.971), with low estimated between-study sensitivity–specificity correlation (0.03). Separate univariate random-effects estimates were similar: sensitivity 0.909 (95% CI 0.873–0.936; *I*^2^ = 42.5%) and specificity 0.944 (95% CI 0.892–0.972; *I*^2^ = 88.7%). Table 2 summarizes the primary bivariate estimate and support analyses; Fig. 1 shows study-specific sensitivity estimates and Fig. 2 shows study-specific specificity estimates. The specificity estimate should be interpreted cautiously because heterogeneity was substantial, likely reflecting timing, patient spectrum, endoleak subtype mix, technology differences, and the use of CTA as an imperfect reference.

**Table 2:** Pooled CEUS apparent diagnostic performance

Analysis	*k*	Paired exams	Sensitivity (95% CI)	*I*^2^ sens	Specificity (95% CI)	*I*^2^ spec
Bivariate primary model	18	2452	0.905 (0.869–0.932)	N/A	0.943 (0.890–0.971)	N/A
Broad all-endoleak univariate support	18	2452	0.909 (0.873–0.936)	42.5%	0.944 (0.892–0.972)	88.7%
Reported interval ≤ 30 days	12	2026	0.914 (0.867–0.945)	57.3%	0.957 (0.896–0.983)	91.9%
CTA-only reference	16	2319	0.907 (0.866–0.936)	46.4%	0.940 (0.881–0.971)	89.7%
Low repetition risk	11	1484	0.925 (0.879–0.954)	44.0%	0.920 (0.864–0.955)	71.4%
Type-II-only Curti 2022 (secondary single-study estimate)	1	119	0.907 (0.796–0.961)	N/A	0.992 (0.890–1.000)	N/A

Curti 2022 is shown only as a secondary type-II-only single-study estimate. It is not part of the 18-study all-endoleak primary pool

### Sensitivity analyses

Restricting the primary analysis to studies with reported CEUS-reference intervals ≤ 30 days did not materially change the sensitivity estimate and improved the pooled specificity point estimate. Restricting to CTA-only reference studies also yielded similar estimates, suggesting that the contemporary composite-reference studies did not drive the pooled result.

The low-repetition-risk sensitivity analysis yielded sensitivity 0.925 and specificity 0.920. The point estimates remained high, while heterogeneity in specificity decreased from 88.7 to 71.4%. This supports the robustness of the overall direction of effect but confirms that repeated examinations per patient and older small cohorts should be treated as major limitations.

Leave-one-out analysis identified Ten Bosch et al. [23] as the main contributor to specificity heterogeneity: excluding it increased specificity to 0.946 and reduced specificity *I*^2^ from 88.7 to 71.5%. Excluding abstract-level reconstructed studies yielded sensitivity 0.911 (95% CI 0.868–0.941) and specificity 0.957 (95% CI 0.901–0.982), indicating that abstract-level inclusion did not drive the results. A separate open-access-only analysis was not prespecified because public availability of the source page is not equivalent to methodological quality or extractability. Deeks-type funnel asymmetry regression did not suggest small-study asymmetry (*p* = 0.907), but this analysis is exploratory with 18 studies [17].

**Fig. 3:**
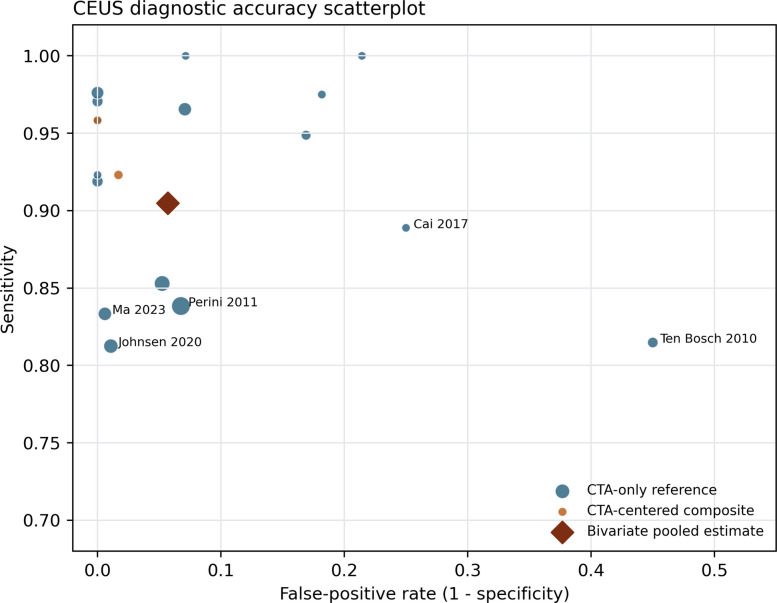
Diagnostic accuracy scatterplot with bivariate pooled estimate

Figure 3 displays the diagnostic accuracy scatterplot. Each study is plotted by its sensitivity–specificity pair, circle size is proportional to the number of paired examinations, and the diamond represents the bivariate pooled estimate.

### Paired discordance

**Fig. 4:**
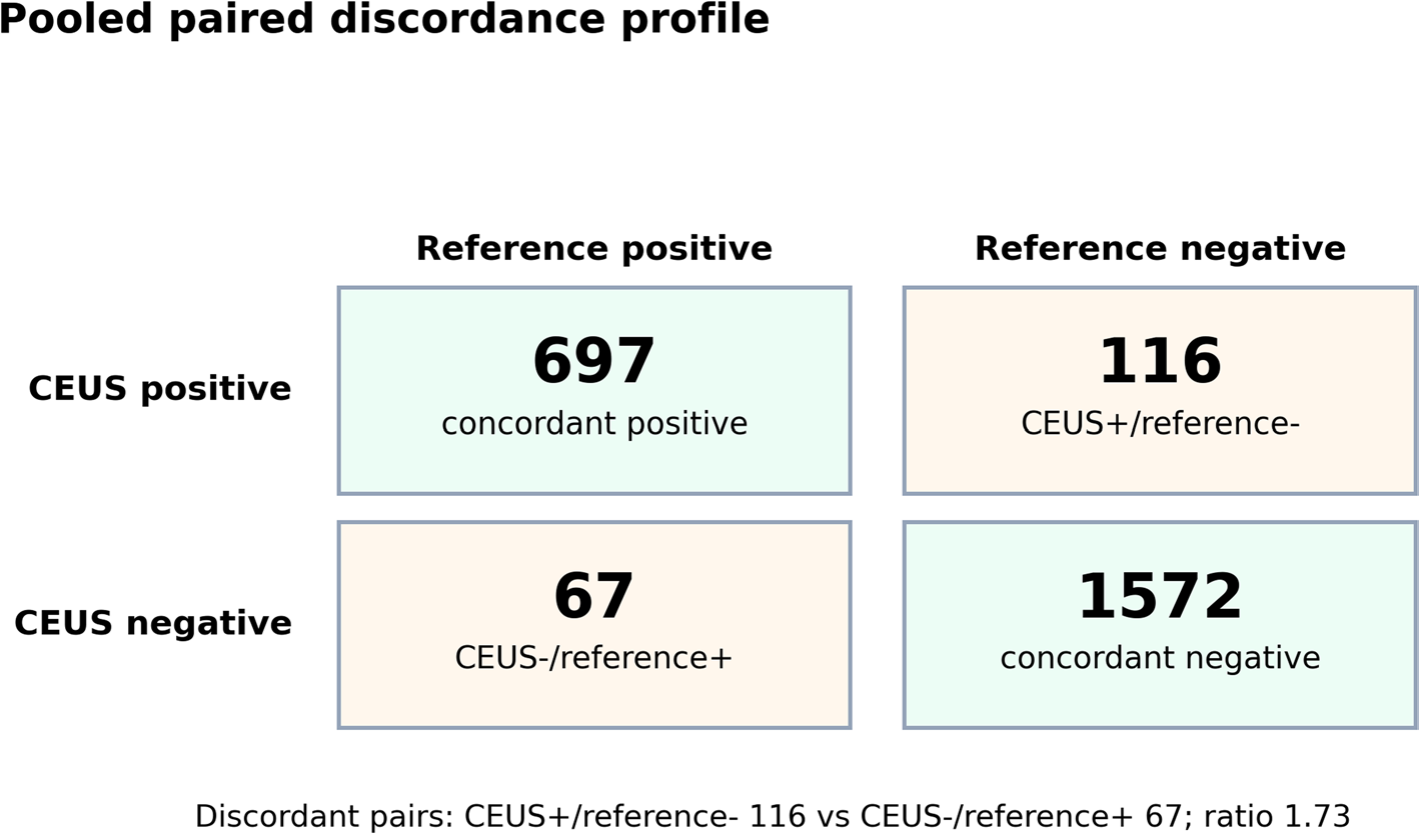
Pooled paired discordance profile using CTA or CTA-centered reference standards. Counts represent paired examinations, not independent patients. Discordant cells should be interpreted as reference-discordant findings rather than definitive modality errors

In the broad analysis, CEUS-positive/reference-negative discordance occurred in 116 paired observations, while CEUS-negative/reference-positive discordance occurred in 67 (Fig. 4). The discordance ratio was 1.73 and the two-sided binomial p value was 0.00036. In the ≤ 30-day sensitivity analysis, the corresponding counts were 97 versus 55.

This imbalance is clinically important but should not be overinterpreted. Under a CTA-centered reference convention, CEUS-positive/reference-negative observations are counted as false positives. However, some may represent true low-flow or delayed endoleaks missed by CTA. Conversely, CEUS-negative/reference-positive observations may reflect bowel gas, high BMI, acoustic limitations, or genuine CEUS misses. The correct inference is that discordant CEUS and reference findings should trigger adjudication or follow-up, not that either modality is automatically wrong.

### Contemporary narrative evidence

Morell-Hofert et al. included 101 patients and reported initial CEUS sensitivity 91.2%, specificity 100%, PPV 100%, and NPV 84.6%, with 44 endoleaks detected by initial CEUS or CTA [11]. Because the accessible data did not provide a simple reconstructible 2 × 2 table compatible with the primary pool, the study was retained narratively.

Jagdeesh et al. reported a 2026 United States cohort of 38 patients with 41 CEUS-CTA pairs within 30 days in a selected population with sac expansion [12]. CEUS and CTA agreed in 27 of 41 pairs; CEUS provided superior diagnostic classification in 13 of 41 pairs and similar diagnosis in 27 of 41 pairs. CEUS sensitivity and specificity were reported as 97.14% and 100%, whereas CTA sensitivity and specificity were 78.57% and 46.15% using the study's adjudication framework. This study is highly relevant to the argument that CTA is imperfect, but the cohort selection and adjudicated reference standard make it unsuitable for pooling with simple CTA-reference studies.

Benedetto et al. supported CEUS-centered surveillance by reporting CEUS sensitivity of 100% versus DUS sensitivity of 75% for any endoleak, and type II sensitivity 93.2% with specificity 99.3% [13]. Frenzel et al. emphasized artifact-aware CEUS interpretation and reported that CEUS was particularly useful for late type II endoleaks and post-embolization cases in which CTA beam-hardening artifacts can be limiting [14]. Panagrosso et al. were a small pilot in preemptive sac coiling and support the concept that CEUS may be helpful when metallic coil artifact limits CTA, but it is not generalizable to routine surveillance [15].

The institutional audit identified several additional nonpoolable but useful records. Park YJ et al. evaluated early postoperative CE-DUS against CTA for overall EVAR-related complications, supporting individualized CEUS-based early surveillance in low-risk patients but not providing an endoleak-specific 2× 2 table [36]. Mauro et al. reported high CEUS-CTA concordance by endoleak type across 220 examinations, but the available abstract did not provide marginal counts for reconstruction [37]. Ghabili et al. compared CEUS and CTA against conventional angiography in a suspected type II endoleak/interventional population, supporting CEUS as a useful rule-out and triage tool but changing the estimand away from routine surveillance [38].

Snyder et al. reported three complex endoleak cases in which intraoperative CEUS helped localize occult or complex endoleaks, guide treatment, provide real-time treatment feedback, and reduce iodinated contrast exposure in a patient with advanced renal disease [16]. This evidence is treatment-focused and not poolable, but it supports the clinical-actionability argument that CEUS may add value beyond binary surveillance detection in selected complex cases.

## Discussion

### Principal findings

This reference-standard-aware synthesis found that CEUS has high apparent diagnostic performance for endoleak detection after EVAR. As detailed in Table 2, the broad pooled sensitivity was approximately 0.91 and pooled specificity approximately 0.94. Sensitivity analyses across the subgroups summarized in Table 1, including short imaging intervals, CTA-only reference standards, and low repetition risk studies, did not materially alter this conclusion. The exploratory Deeks-type regression did not suggest small-study asymmetry, although this result should be interpreted cautiously because the analysis included only 18 studies.

A central interpretive finding is the discordance profile rather than any single pooled value. As shown in Fig. 4, CEUS-positive/reference-negative discordance was more common than CEUS-negative/reference-positive discordance. In a conventional CTA-reference diagnostic accuracy table, this decreases CEUS specificity. Clinically, however, some CEUS-positive/reference-negative findings may represent the known strength of CEUS: dynamic visualization of slow, delayed, or intermittent endoleak flow not captured by CTA-centered reference imaging. This is especially plausible for type II endoleaks and post-embolization settings. The result therefore supports a practical surveillance message: CEUS and CTA are complementary, and discordance should be adjudicated in the context of sac behavior, endoleak type, renal function, body habitus, and whether intervention is being planned.

### Why not claim that CEUS replaces CTA?

Several included studies have used replacement language [11, 13, 14, 25], and prior reviews reported high CEUS performance without foregrounding CTA-reference imperfection, clustering, and discordance directionality [4, 5]. The present synthesis does not support an unrestricted replacement claim. CTA remains important for endograft structure, migration, component separation, sealing zones, branch anatomy, procedural planning, and evaluation when CEUS is limited by acoustic factors. CEUS is better framed as a CTA-sparing or CTA-triaging tool in selected surveillance pathways.

A clinically defensible pathway is proposed below as an evidence-aligned synthesis rather than a formally validated decision model, with each step anchored to the included evidence and to current guidelines:

- Use CTA early after EVAR or when structural assessment is required, consistent with ESVS 2024 and ESC 2024 recommendations that retain cross-sectional imaging for baseline and structural evaluation [1, 2].
- Use CEUS as a high-sensitivity surveillance test when renal function, cumulative radiation, or low-flow endoleak detection is a priority; this is supported by the pooled sensitivity of approximately 0.91 and by an included study reporting CEUS detection of type II endoleaks during EVAR follow-up (Johnsen [20]), and by an activity-based cost analysis reporting lower cost than CTA in this setting (Faccioli [26]).
- Use CTA, MRA, DSA, or multidisciplinary adjudication when CEUS and CTA are discordant, when sac enlargement occurs without a clear endoleak, or when type I/III endoleak is suspected; this reflects both the imperfect-reference and discordance findings of the present review and the structural and procedural-planning role of cross-sectional imaging emphasized in guidelines [1–3].

This framing aligns with contemporary guideline logic, which recognizes CT, DUS, and CEUS as major follow-up imaging modalities rather than mutually exclusive alternatives [1–3].

### Reference-standard limitations

The main methodological limitation is the conventional use of CTA as the reference standard. CTA is excellent for structural anatomy but can miss delayed enhancement depending on acquisition phase and may be impaired by metallic or embolization artifacts. A static or limited-phase CTA can be less sensitive to low-flow type II endoleaks than a real-time CEUS examination. Therefore, pooled CEUS specificity against CTA may underestimate the clinical specificity of CEUS in some settings.

The reverse is also true: CEUS is not immune to false-positive interpretation. Artifacts, bowel gas, patient habitus, and operator experience matter. The appropriate solution is not to declare either modality the universal truth standard, but to build future studies around composite adjudication: independent blinded CTA and CEUS interpretation, DSA when clinically performed, longitudinal sac behavior, reintervention findings, and prespecified follow-up rules.

### Clinical actionability of endoleak subtypes

The primary endpoint was any endoleak, because that was the most consistently reconstructible endpoint across studies. This endpoint is clinically useful for surveillance sensitivity, but it should not be confused with proof of equivalent performance for all clinically urgent endoleak subtypes. Type I and III endoleaks require structural localization and procedural planning, areas in which CTA or another cross-sectional angiographic modality remains central. Type II endoleaks are the setting in which CEUS is most plausibly advantaged by dynamic real-time flow assessment, especially when CTA is single-phase, delayed enhancement is present, or embolization artifacts are present.

### Heterogeneity

Heterogeneity in sensitivity was moderate; heterogeneity in specificity was high. This dispersion is visually evident in Fig. 2, where confidence intervals and point estimates vary more than in the sensitivity forest plot (Fig. 1). Several factors likely contributed:

- Older versus newer CEUS equipment and contrast-specific software.
- Differences in CTA protocols, especially arterial-only versus multiphasic/delayed imaging.
- Variation in CEUS-CTA timing.
- Mixture of first surveillance, annual surveillance, sac expansion cohorts, and post-intervention cases.
- Aggregation of type I, II, III, and mixed endoleaks into a single endpoint.
- Repeated examinations per patient, which can inflate precision and correlate results.
- Spectrum bias from mixing routine surveillance cohorts with sac-expansion, post-intervention, and selected referral cohorts.
- Threshold effects related to CEUS interpretation criteria, CTA phase timing, and local definitions of clinically relevant flow.

Because subtype-level 2 × 2 data were not consistently available, the present review does not pool type I/III separately from type II. Descriptively, the included and contemporary nonpoolable evidence suggests CEUS is particularly valuable for type II endoleaks, whereas CTA retains a structural advantage for type I/III planning. A subtype-specific meta-analysis should be performed only if full-text extraction yields at least eight studies with comparable subtype-level 2 × 2 data.

### Clinical interpretation

The results support CEUS as a robust surveillance modality in experienced hands. In stable patients after EVAR, especially those with renal dysfunction, contrast allergy risk, or high cumulative imaging burden, CEUS can reduce CTA exposure. CEUS may also be the more informative test when sac enlargement persists with negative or equivocal CTA.

For high-risk endoleaks, especially type I and III, CEUS should not be used as an isolated gatekeeper when intervention planning depends on graft anatomy. In those scenarios, CTA or another cross-sectional angiographic modality remains essential.

### Strengths

This review avoids quality-score-weighted pooling, which is not appropriate for diagnostic test accuracy synthesis, and separates poolable DTA evidence from narrative evidence with incompatible reference standards. It adds contemporary studies through the 13 May 2026 lock-date search, adds a CAPES/UNB Embase and Scopus institutional coverage audit, explicitly audits clustering and reference-standard class, and treats CTA as an imperfect comparator rather than a gold standard. The institutional audit changed the review constructively: it added one poolable abstract-level study, prompted re-adjudication of older public records that were previously underused, and documented several nonpoolable but clinically relevant studies. The analysis is reproducible from project-level CSV files, scripts, figures, and the supplementary data package.

### Limitations

Several limitations should be considered. First, many studies did not fully report blinding between CEUS and CTA readers, and CTA itself is an imperfect reference standard. Second, Embase and Scopus were audited through institutional access, but Web of Science export was not completed at the lock date; this was mitigated by citation checking of prior reviews, included studies, and contemporary full texts where available. Third, screening and extraction used a single-reviewer structured verification workflow rather than fully duplicated independent screening of every record, which is a recognized potential source of selection and extraction bias; to mitigate this and support reproducibility, every included 2 × 2 table was re-checked against its source report, abstract, or documented reconstruction basis (Supplementary Table S5), a source-basis sensitivity analysis excluding abstract-level reconstructions left the estimates essentially unchanged (sensitivity 0.911, specificity 0.957), and all screening outputs, extraction tables, and analysis scripts are shared to allow independent re-execution; these measures improve auditability and reduce extraction error but do not substitute for fully duplicated independent screening. Fourth, several older or conference-derived studies relied on abstract-level or citation-audit 2 × 2 reconstruction rather than directly reported full 2 × 2 tables; source-basis sensitivity excluding abstract-level reconstructed studies was reassuring. Fifth, repeated paired examinations per patient were common: 7 studies contributed 968 of the 2452 paired examinations (39.5%), and patient-level data were unavailable for cluster-adjusted modeling, so within-study confidence intervals in these cohorts are likely too narrow and the pooled precision is correspondingly overstated. In the prespecified low-repetition-risk analysis the point estimates remained broadly similar (sensitivity 0.925, specificity 0.920, with specificity *I*^2^ falling from 88.7 to 71.4%), making the main qualitative conclusion unlikely to be explained by clustering alone, although residual effects on precision and on the pooled estimates cannot be excluded. Sixth, the pooled estimates are apparent performance against CTA-centered references, not adjudicated clinical truth. Seventh, specificity heterogeneity was high and decreased only partially in sensitivity analyses. Eighth, subtype-level comparative accuracy could not be robustly pooled from the current extraction.

These limitations do not negate the central finding that CEUS is high-performing, but they require conservative wording and support the decision to frame CEUS as CTA-sparing and adjudicative rather than as a universal CTA replacement.

## Conclusions

CEUS demonstrates high apparent sensitivity and specificity for endoleak detection after EVAR when compared with CTA or CTA-centered reference imaging. The discordance profile, with significantly more CEUS-positive/reference-negative pairs than the reverse, suggests that some apparent false-positive CEUS findings may represent low-flow or delayed endoleaks not captured by CTA-centered reference imaging. However, substantial specificity heterogeneity and the inherent limitations of CTA as an imperfect reference standard argue against an unqualified claim that CEUS universally replaces CTA. The most evidence-consistent interpretation is that CEUS should be integrated as a CTA-sparing surveillance and adjudication tool within structured post-EVAR pathways, with CTA retained for early baseline assessment, structural evaluation, procedural planning, and discordant or high-risk findings.

## Supplementary Information

Supplementary Material 1: Supplementary Methods S1. Search Strategy. Supplementary Methods S2. Lock-Date Search Summary. Supplementary Table S1. Study-Level Diagnostic Extraction. Supplementary Table S2. Primary-Pool 2 × 2 Diagnostic Tables. Supplementary Table S3. Reference-Standard and Unit-of-Analysis Audit. Supplementary Table S4. Final QUADAS-2 and QUADAS-C Mapping. Supplementary Table S5. Source Verification Matrix. Supplementary Table S6. Contemporary Narrative Evidence. Supplementary Table S7. Bivariate, Source-Basis, and Small-Study-Effect Analyses. Supplementary Table S8. Leave-One-Out Analysis. Supplementary Figure S1: PRISMA-DTA public lock-date flow diagram

## Data Availability

All data generated or analyzed during this study are included in this article, its supplementary information files, and the supplementary data package prepared for submission. The package contains extraction tables, search metadata, screening outputs, risk-of-bias tables, analysis outputs, figures, and analysis scripts.

## Abbreviations

AAA: Abdominal aortic aneurysm
CEUS: Contrast-enhanced ultrasound
CI: Confidence interval
CTA: Computed tomography angiography
DSA: Digital subtraction angiography
DUS: Duplex ultrasound
EVAR: Endovascular aneurysm repair
MRA: Magnetic resonance angiography
PRISMA-DTA: Preferred Reporting Items for a Systematic Review and Meta-analysis of Diagnostic Test Accuracy Studies
PRISMA-S: Preferred Reporting Items for Systematic Reviews and Meta-Analyses literature search extension
QUADAS-2: Quality Assessment of Diagnostic Accuracy Studies 2
QUADAS-C: Quality Assessment of Comparative Diagnostic Accuracy Studies
SMI: Superb microvascular imaging
