# Supplementary material for: Contrast-enhanced ultrasound versus CT angiography for endoleak detection after EVAR: a reference-standard-aware systematic review and meta-analysis
Source: CVIR Endovasc. 2026 Jul 22;9:90. doi: 10.1186/s42155-026-00731-6 (PMC13391992; doi:10.1186/s42155-026-00731-6)

**Supplementary Methods and Tables v1.3**

Version note: this v1.3 supplement preserves the locked search, extraction, risk-of-bias, and analysis tables from the submission-prepared package and aligns the file naming with the final submission package dated 18 May 2026.

# Supplementary Methods S1. Search Strategy

## Search Strategy Master v1.0

Project: CEUS versus CTA for endoleak detection after infrarenal EVAR

Initial search freeze: 2026-05-12

Public lock-date re-run: 2026-05-13

Public lock-date re-run and institutional audit: 2026-05-13

Mandatory rerun: completed on 2026-05-13 before extraction lock; Web of Science export was not completed because access/export verification could not be resolved at the lock date.

## Core Concepts

1. Disease/procedure:

- abdominal aortic aneurysm

- EVAR

- endovascular aneurysm repair

- aortic endograft

- aortic stent graft

1. Target condition:

- endoleak

- endoleaks

1. Index/comparator tests:

- contrast-enhanced ultrasound

- CEUS

- contrast enhanced ultrasound

- microbubble

- SonoVue

- Lumason

- computed tomography angiography

- CTA

- CT angiography

- angiotomography

## PubMed/MEDLINE

(

"Endoleak"[Mesh] OR endoleak*[tiab]

)

AND

(

"Ultrasonography"[Mesh] OR "contrast-enhanced ultrasound"[tiab] OR

"contrast enhanced ultrasound"[tiab] OR CEUS[tiab] OR microbubble*[tiab] OR

SonoVue[tiab] OR Lumason[tiab]

)

AND

(

"Tomography, X-Ray Computed"[Mesh] OR "computed tomography angiography"[tiab] OR

"CT angiography"[tiab] OR CTA[tiab] OR "computed tomography"[tiab] OR

angiotomograph*[tiab]

)

AND

(

"Endovascular Procedures"[Mesh] OR "Aortic Aneurysm, Abdominal"[Mesh] OR

EVAR[tiab] OR "endovascular aneurysm repair"[tiab] OR

"endovascular aortic aneurysm repair"[tiab] OR "aortic endograft"[tiab] OR

"aortic stent graft"[tiab] OR "stent-graft"[tiab]

)

AND ("2000/01/01"[Date - Publication] : "3000"[Date - Publication])

## Embase

('endoleak'/exp OR endoleak*:ti,ab)

AND

('contrast enhanced ultrasound'/exp OR 'ultrasonography'/exp OR

'contrast-enhanced ultrasound':ti,ab OR 'contrast enhanced ultrasound':ti,ab OR

ceus:ti,ab OR microbubble*:ti,ab OR sonovue:ti,ab OR lumason:ti,ab)

AND

('computed tomographic angiography'/exp OR 'computed tomography'/exp OR

'computed tomography angiography':ti,ab OR 'ct angiography':ti,ab OR

cta:ti,ab OR angiotomograph*:ti,ab)

AND

('endovascular aneurysm repair'/exp OR 'abdominal aortic aneurysm'/exp OR

evar:ti,ab OR 'endovascular aneurysm repair':ti,ab OR

'endovascular aortic aneurysm repair':ti,ab OR 'aortic endograft':ti,ab OR

'aortic stent graft':ti,ab OR 'stent graft':ti,ab)

AND [2000-2026]/py

## Scopus

TITLE-ABS-KEY(endoleak*)

AND TITLE-ABS-KEY("contrast-enhanced ultrasound" OR "contrast enhanced ultrasound"

OR CEUS OR microbubble* OR SonoVue OR Lumason)

AND TITLE-ABS-KEY("computed tomography angiography" OR "CT angiography" OR CTA

OR "computed tomography" OR angiotomograph*)

AND TITLE-ABS-KEY(EVAR OR "endovascular aneurysm repair"

OR "endovascular aortic aneurysm repair"

OR "aortic endograft" OR "aortic stent graft" OR "stent graft")

AND PUBYEAR > 1999

## Web of Science Core Collection

TS=(endoleak*)

AND TS=("contrast-enhanced ultrasound" OR "contrast enhanced ultrasound" OR CEUS

OR microbubble* OR SonoVue OR Lumason)

AND TS=("computed tomography angiography" OR "CT angiography" OR CTA

OR "computed tomography" OR angiotomograph*)

AND TS=(EVAR OR "endovascular aneurysm repair"

OR "endovascular aortic aneurysm repair"

OR "aortic endograft" OR "aortic stent graft" OR "stent graft")

Timespan: 2000-2026

## Cochrane Library

(endoleak*):ti,ab,kw

AND ("contrast-enhanced ultrasound" OR "contrast enhanced ultrasound" OR CEUS

OR microbubble* OR SonoVue OR Lumason):ti,ab,kw

AND ("computed tomography angiography" OR "CT angiography" OR CTA

OR "computed tomography" OR angiotomograph*):ti,ab,kw

AND (EVAR OR "endovascular aneurysm repair"

OR "endovascular aortic aneurysm repair"

OR "aortic endograft" OR "aortic stent graft" OR "stent graft"):ti,ab,kw

Publication year: 2000-2026

## Public API Searches Used For Reproducible Pre-Screening

Publicly executable searches:

- PubMed/MEDLINE via NCBI E-utilities.
- Europe PMC.
- Crossref.
- OpenAlex.

These public searches provide a reproducible broad corpus for initial screening

and deduplication. A CAPES/UNB institutional audit was subsequently completed

in Embase and Scopus on 2026-05-13.

## Institutional Search Audit Completed on 2026-05-13

- Embase via CAPES/UNB: 1,222 records exported in RIS across batches and recombined in `03_search/institutional_2026-05-13/raw_exports/embase_all_1222_combined.ris`.
- Scopus via CAPES/UNB: 142 result records captured from the institutional results table in `03_search/institutional_2026-05-13/raw_exports/scopus_2026-05-13_scraped_142.csv`; account-level export was unavailable at the lock date.
- Web of Science Core Collection via CAPES/UNB: access was confirmed and the advanced-search page was opened, but result execution/export could not be completed before the lock date because platform verification was not resolved.
- Institutional deduplication output: `03_search/deduplication/2026-05-13_institutional_dedup.csv`.
- Institutional title/abstract candidates not found in the public search: `04_screening/title_abstract/2026-05-13_institutional_new_candidates_not_in_public.csv`.

# Supplementary Methods S2. Lock-Date Search Summary

The public lock-date search was run on 13 May 2026 in PubMed/MEDLINE, Europe PMC, Crossref, and OpenAlex, and was followed by a CAPES/UNB institutional audit in Embase and Scopus on the same date. The public search identified 986 raw records and the institutional audit identified 1,364 additional raw database/result records (Embase 1,222; Scopus 142), for 2,350 total raw records. After deduplication across sources, 1,654 unique records remained. Structured title/abstract prioritization retained 185 records for relevance assessment. The final synthesis retained 18 all-endoleak quantitative studies, one type-II-only secondary study, and 15 narrative/context studies. Web of Science Core Collection was opened through CAPES/UNB, but records could not be exported before the lock date because access/export verification was not completed.

# Supplementary Table S1. Study-Level Diagnostic Extraction

| **first_author** | **year** | **participants** | **paired_exams** | **interval_ceus_cta** | **reference_standard** | **scope** | **ceus_tp** | **ceus_fn** | **ceus_fp** | **ceus_tn** | **included_main_quant** |
| --- | --- | --- | --- | --- | --- | --- | --- | --- | --- | --- | --- |
| Motta | 2012 | 88 | 142 | Same day | CTA | All endoleaks | 34 | 3 | 0 | 105 | yes |
| Perini | 2011 | 395 | 395 | <15 days | CTA | All endoleaks | 83 | 16 | 20 | 276 | yes |
| Johnsen | 2020 | 92 | 233 | Same day | CTA | All endoleaks | 39 | 9 | 2 | 183 | yes |
| Park | 2022 | 110 | 110 | <3 months | CTA | All endoleaks | 37 | 2 | 12 | 59 | yes |
| Gurtler | 2013 | 171 | 200 | 0-30 days | CTA | All endoleaks | 84 | 3 | 8 | 105 | yes |
| Ten Bosch | 2010 | 83 | 127 | 0-2 days | CTA | All endoleaks | 22 | 5 | 45 | 55 | yes |
| Houdek | 2015 | 16 | 28 | Not reported | CTA | All endoleaks | 12 | 1 | 0 | 15 | yes |
| Bredahl | 2016 | 278 | 278 | 0-7 days | CTA | All endoleaks | 58 | 10 | 11 | 199 | yes |
| Faccioli | 2018 | 137 | 137 | 2-7 days | CTA | All endoleaks | 99 | 3 | 0 | 35 | yes |
| Clevert | 2008 | 43 | 43 | 1 day | CTA | All endoleaks | 15 | 0 | 2 | 26 | yes |
| Henao | 2006 | 20 | 20 | Same surveillance episode; exact... | CTA | All endoleaks | 6 | 0 | 3 | 11 | yes |
| Iezzi | 2009 | 84 | 84 | Same day | CTA | All endoleaks | 39 | 1 | 8 | 36 | yes |
| David | 2016 | 181 | 181 | 0-6 days | CTA | All endoleaks | 41 | 1 | 0 | 139 | yes |
| Cantisani | 2011 | 108 | 108 | 0-7 days | CTA | All endoleaks | 23 | 1 | 0 | 84 | yes |
| Cai | 2017 | 30 | 30 | Within 3 months | CTA | All endoleaks | 16 | 2 | 3 | 9 | yes |
| Ma | 2023 | 102 | 203 | Not reported in abstract | CTA | All endoleaks | 30 | 6 | 1 | 166 | yes |
| Wang | 2024 | 98 | 98 | Within 1 month | CTA/DSA composite | All endoleaks | 36 | 3 | 1 | 58 | yes |
| Nijhawan | 2024 | 28 | 35 | Not stated in abstract | CTA/conventional angiography | All endoleaks | 23 | 1 | 0 | 11 | yes |
| Curti | 2022 | 119 | 119 | CTA at 3 months and CEUS at 4 months | CTA | Type II endoleaks only | 49 | 5 | 0 | 65 | no |

# Supplementary Table S2. Primary-Pool 2x2 Diagnostic Tables

| **first_author** | **year** | **participants** | **paired_exams** | **reference_standard** | **source_basis** | **ceus_tp** | **ceus_fn** | **ceus_fp** | **ceus_tn** |
| --- | --- | --- | --- | --- | --- | --- | --- | --- | --- |
| Motta | 2012 | 88 | 142 | CTA | Dissertation tables 1-3 | 34 | 3 | 0 | 105 |
| Perini | 2011 | 395 | 395 | CTA | Dissertation tables 1-3 | 83 | 16 | 20 | 276 |
| Johnsen | 2020 | 92 | 233 | CTA | Dissertation tables 1-3 | 39 | 9 | 2 | 183 |
| Park | 2022 | 110 | 110 | CTA | Dissertation tables 1-3 | 37 | 2 | 12 | 59 |
| Gurtler | 2013 | 171 | 200 | CTA | Dissertation tables 1-3 | 84 | 3 | 8 | 105 |
| Ten Bosch | 2010 | 83 | 127 | CTA | Dissertation tables 1-3 | 22 | 5 | 45 | 55 |
| Houdek | 2015 | 16 | 28 | CTA | Dissertation tables 1-3 | 12 | 1 | 0 | 15 |
| Bredahl | 2016 | 278 | 278 | CTA | Dissertation tables 1-3 | 58 | 10 | 11 | 199 |
| Faccioli | 2018 | 137 | 137 | CTA | Dissertation tables 1-3 | 99 | 3 | 0 | 35 |
| Clevert | 2008 | 43 | 43 | CTA | Dissertation tables 1-3 | 15 | 0 | 2 | 26 |
| Henao | 2006 | 20 | 20 | CTA | PubMed abstract | 6 | 0 | 3 | 11 |
| Iezzi | 2009 | 84 | 84 | CTA | PubMed abstract | 39 | 1 | 8 | 36 |
| David | 2016 | 181 | 181 | CTA | Dissertation tables 1-3 | 41 | 1 | 0 | 139 |
| Cantisani | 2011 | 108 | 108 | CTA | Dissertation tables 1-3 | 23 | 1 | 0 | 84 |
| Cai | 2017 | 30 | 30 | CTA | Embase abstract | 16 | 2 | 3 | 9 |
| Ma | 2023 | 102 | 203 | CTA | PubMed abstract | 30 | 6 | 1 | 166 |
| Wang | 2024 | 98 | 98 | CTA/DSA composite | QIMS full text | 36 | 3 | 1 | 58 |
| Nijhawan | 2024 | 28 | 35 | CTA/conventional angiography | Sage abstract | 23 | 1 | 0 | 11 |

# Supplementary Table S3. Reference-Standard and Unit-of-Analysis Audit

| **first_author** | **year** | **exam_patient_ratio** | **reference_class** | **subtype_2x2_available** | **clustering_risk** | **primary_pool_status** |
| --- | --- | --- | --- | --- | --- | --- |
| Motta | 2012 | 1.6136363636363635 | CTA only | no comparable subtype- level 2x2... | high | included in broad all- endoleak pool |
| Perini | 2011 | 1.0 | CTA only | no comparable subtype- level 2x2... | low | included in broad all- endoleak pool |
| Johnsen | 2020 | 2.532608695652174 | CTA only | no comparable subtype- level 2x2... | high | included in broad all- endoleak pool |
| Park | 2022 | 1.0 | CTA only | no comparable subtype- level 2x2... | low | included in broad all- endoleak pool |
| Gurtler | 2013 | 1.1695906432748537 | CTA only | no comparable subtype- level 2x2... | moderate | included in broad all- endoleak pool |
| Ten Bosch | 2010 | 1.5301204819277108 | CTA only | no comparable subtype- level 2x2... | high | included in broad all- endoleak pool |
| Houdek | 2015 | 1.75 | CTA only | no comparable subtype- level 2x2... | high | included in broad all- endoleak pool |
| Bredahl | 2016 | 1.0 | CTA only | no comparable subtype- level 2x2... | low | included in broad all- endoleak pool |
| Faccioli | 2018 | 1.0 | CTA only | no comparable subtype- level 2x2... | low | included in broad all- endoleak pool |
| Clevert | 2008 | 1.0 | CTA only | no comparable subtype- level 2x2... | low | included in broad all- endoleak pool |
| Henao | 2006 | 1.0 | CTA only | no comparable subtype- level 2x2... | low | included in broad all- endoleak pool |
| Iezzi | 2009 | 1.0 | CTA only | no comparable subtype- level 2x2... | low | included in broad all- endoleak pool |
| David | 2016 | 1.0 | CTA only | no comparable subtype- level 2x2... | low | included in broad all- endoleak pool |
| Cantisani | 2011 | 1.0 | CTA only | no comparable subtype- level 2x2... | low | included in broad all- endoleak pool |
| Cai | 2017 | 1.0 | CTA only | no comparable subtype- level 2x2... | low | included in broad all- endoleak pool |
| Ma | 2023 | 1.9901960784313726 | CTA only | no comparable subtype- level 2x2... | high | included in broad all- endoleak pool |
| Wang | 2024 | 1.0 | CTA-centered composite/adjudicated | no comparable subtype- level 2x2... | low | included in broad all- endoleak pool |
| Nijhawan | 2024 | 1.25 | CTA-centered composite/adjudicated | no comparable subtype- level 2x2... | moderate | included in broad all- endoleak pool |
| Curti | 2022 | 1.0 | CTA only | type II only | low | type- II-only secondary/narrative |

# Supplementary Table S4. Final QUADAS-2 and QUADAS-C Mapping

| **first_author** | **year** | **patient_selection_risk** | **index_test_risk** | **reference_standard_risk** | **flow_timing_risk** | **applicability_concerns** | **quadas_c_comparative_comment** |
| --- | --- | --- | --- | --- | --- | --- | --- |
| Motta | 2012 | unclear | unclear | unclear | low | low | CTA-only comparator; CTA imperfection... |
| Perini | 2011 | unclear | unclear | unclear | low | low | CTA-only comparator; CTA imperfection... |
| Johnsen | 2020 | low | unclear | unclear | low | low | CTA-only comparator; CTA imperfection... |
| Park | 2022 | unclear | unclear | unclear | high | moderate | CTA-only comparator; CTA imperfection... |
| Gurtler | 2013 | unclear | unclear | unclear | low | low | CTA-only comparator; CTA imperfection... |
| Ten Bosch | 2010 | unclear | unclear | unclear | low | moderate | CTA-only comparator; CTA imperfection... |
| Houdek | 2015 | unclear | unclear | unclear | unclear | moderate | CTA-only comparator; CTA imperfection... |
| Bredahl | 2016 | low | unclear | unclear | low | low | CTA-only comparator; CTA imperfection... |
| Faccioli | 2018 | unclear | unclear | unclear | low | low | CTA-only comparator; CTA imperfection... |
| Clevert | 2008 | unclear | unclear | unclear | low | moderate | CTA-only comparator; CTA imperfection... |
| Henao | 2006 | unclear | unclear | unclear | unclear | moderate | CTA-only comparator; CTA imperfection... |
| Iezzi | 2009 | unclear | unclear | unclear | low | low | CTA-only comparator; CTA imperfection... |
| David | 2016 | unclear | unclear | unclear | low | low | CTA-only comparator; CTA imperfection... |
| Cantisani | 2011 | low | unclear | unclear | low | low | CTA-only comparator; CTA imperfection... |
| Cai | 2017 | high | unclear | unclear | high | moderate | CTA-only comparator; CTA imperfection... |
| Ma | 2023 | unclear | unclear | unclear | unclear | low | CTA-only comparator; CTA imperfection... |
| Wang | 2024 | unclear | unclear | unclear | low | moderate | CTA-centered composite/adjudicated... |
| Nijhawan | 2024 | high | unclear | unclear | unclear | moderate | CTA-centered composite/adjudicated... |
| Curti | 2022 | unclear | unclear | unclear | high | moderate | CTA-only comparator; CTA imperfection... |

# Supplementary Table S5. Source Verification Matrix

| **first_author** | **year** | **primary_pool** | **source_basis** | **verification_status_v1** | **pooling_decision_v1** |
| --- | --- | --- | --- | --- | --- |
| Motta | 2012 | yes | Dissertation tables 1-3 | Thesis extraction tables used as the... | primary all- endoleak pool |
| Perini | 2011 | yes | Dissertation tables 1-3 | Thesis extraction tables used as the... | primary all- endoleak pool |
| Johnsen | 2020 | yes | Dissertation tables 1-3 | Thesis extraction tables used as the... | primary all- endoleak pool |
| Park | 2022 | yes | Dissertation tables 1-3 | Thesis extraction tables used as the... | primary all- endoleak pool |
| Gurtler | 2013 | yes | Dissertation tables 1-3 | Thesis extraction tables used as the... | primary all- endoleak pool |
| Ten Bosch | 2010 | yes | Dissertation tables 1-3 | Thesis extraction tables used as the... | primary all- endoleak pool |
| Houdek | 2015 | yes | Dissertation tables 1-3 | Thesis extraction tables used as the... | primary all- endoleak pool |
| Bredahl | 2016 | yes | Dissertation tables 1-3 | Thesis extraction tables used as the... | primary all- endoleak pool |
| Faccioli | 2018 | yes | Dissertation tables 1-3 | Thesis extraction tables used as the... | primary all- endoleak pool |
| Clevert | 2008 | yes | Dissertation tables 1-3 | Thesis extraction tables used as the... | primary all- endoleak pool |
| Henao | 2006 | yes | PubMed abstract | Abstract-level source used for... | primary all- endoleak pool |
| Iezzi | 2009 | yes | PubMed abstract | Abstract-level source used for... | primary all- endoleak pool |
| David | 2016 | yes | Dissertation tables 1-3 | Thesis extraction tables used as the... | primary all- endoleak pool |
| Cantisani | 2011 | yes | Dissertation tables 1-3 | Thesis extraction tables used as the... | primary all- endoleak pool |
| Cai | 2017 | yes | Embase abstract | Abstract-level source used for... | primary all- endoleak pool |
| Ma | 2023 | yes | PubMed abstract | Abstract-level source used for... | primary all- endoleak pool |
| Wang | 2024 | yes | QIMS full text | Public full text/source page used for... | primary all- endoleak pool |
| Nijhawan | 2024 | yes | Sage abstract | Abstract-level source used for... | primary all- endoleak pool |
| Curti | 2022 | no | MDPI full text | Public full text/source page used for... | secondary/narrative, not primary pool |

# Supplementary Table S6. Contemporary Narrative Evidence

| **study_id** | **first_author** | **year** | **country** | **participants** | **paired_exams** | **reason_not_in_main_quant** | **key_findings** | **source_basis** | **notes** |
| --- | --- | --- | --- | --- | --- | --- | --- | --- | --- |
| morell_hofert_2024 | Morell- Hofert | 2024 | Austria | 101 | Not fully reconstructible from abstract | 2x2 not reconstructible from accessible... | 44 endoleaks detected by initial CEUS... | ScienceDirect/PubMed abstract | Important contemporary validation;... |
| jagdeesh_2026 | Jagdeesh | 2026 | USA | 38 | 41 | Composite/adjudicated diagnostic... | CEUS and CTA agreed in 27/41 pairs;... | PMC full text | High- selection cohort with sac... |
| benedetto_2022 | Benedetto | 2022 | Italy | 125 | 228 | Primary comparison CEUS versus DUS;... | CEUS sensitivity 100% versus DUS 75%... | MDPI full text | Supports CEUS-based surveillance... |
| frenzel_2021 | Frenzel | 2021 | Germany | Not extracted | Not extracted | 2x2 not available from accessible abstract | CEUS artifacts occurred frequently but... | ScienceDirect abstract | Important for discussion of CTA... |
| panagrosso_2024 | Panagrosso | 2024 | Denmark | 4 | 4 | Pilot/special population after... | 3D-CEUS detected endoleaks in coil-... | PMC full text | Exclude from main review because pilot,... |
| park_yj_2023 | Park YJ | 2023 | South Korea | 100 | 100 | Overall EVAR- related complications... | CE-DUS and CTA were performed within... | Embase institutional abstract | Relevant to individualized early... |
| mauro_2010 | Mauro | 2010 | Italy | 122 | 220 | Concordance reported by endoleak type... | Prospective CEUS and CTA within two... | Embase institutional abstract | Potentially important if full text... |
| kopp_2008 | Kopp | 2008 | Germany | Not reported | Not reported | No diagnostic 2x2 in available abstract | CEUS was described as a valuable... | Embase institutional abstract | Likely overlaps conceptually with the... |
| hodge_2007 | Hodge | 2007 | USA | Not clearly independent | 18 | Overlapping early report of continuous-... | Continuous Optison infusion with color... | Embase institutional abstract | Not double- counted because Henao 2006... |
| kamal_2008 | Kamal | 2008 | Canada | 51 | 86 | Duplex- focused comparison; contrast... | Duplex ultrasound had moderate... | Embase institutional abstract | Useful as cautionary older evidence;... |
| freyrie_2007 | Freyrie | 2007 | Italy | 16 | 16 | Type-II- only selected cohort | CEUS sensitivity was reported as 100%... | Embase institutional abstract | Retained outside primary all- endoleak... |
| ghabili_2025 | Ghabili | 2025 | USA | Not reported | Not reported | Type-II suspected/interventional cohort... | CEUS and CTA were compared against... | Embase institutional abstract | Clinically relevant but not comparable... |
| cheung_2025 | Cheung | 2025 | Australia | 33 | 33 | Algorithm/characterization study... | CEUS clarified endoleak type after... | Embase institutional abstract | Supports clinical utility and safety... |
| d_alessio_2019 | D'Alessio | 2019 | Italy | 111 | Not applicable | Surveillance pathway report without... | A single- center experience used CEUS-... | Embase institutional abstract | Supports CTA- sparing pathway... |
| snyder_2025 | Snyder | 2025 | USA | 3 cases | Not applicable | Intraoperative/treatment- focused case... | CEUS was used intraoperatively to... | PubMed/PMC full text | Useful for clinical actionability... |

# Supplementary Table S7. Bivariate, Source-Basis, and Small-Study-Effect Analyses

## Bivariate primary model

| **status** | **k** | **sensitivity** | **sens_ci_low** | **sens_ci_high** | **specificity** | **spec_ci_low** | **spec_ci_high** | **tau2_sensitivity_logit** | **tau2_specificity_logit** | **between_study_corr** | **neg_log_likelihood** |
| --- | --- | --- | --- | --- | --- | --- | --- | --- | --- | --- | --- |
| converged | 18 | 0.9049240030133902 | 0.8693310965122111 | 0.9315847528054648 | 0.9431697332202641 | 0.8899254258540968 | 0.9714844257337406 | 0.21356788814236025 | 1.7382825150110508 | 0.03022746777545437 | 54.89961470971517 |

## Source-basis sensitivity

| **analysis** | **k** | **participants** | **paired_exams** | **sensitivity** | **sens_ci_low** | **sens_ci_high** | **specificity** | **spec_ci_low** | **spec_ci_high** | **note** |
| --- | --- | --- | --- | --- | --- | --- | --- | --- | --- | --- |
| broad_all_endoleaks | 18 | 2064 | 2452 | 0.9089846318160639 | 0.8726172790087499 | 0.9357338883589171 | 0.944273317653338 | 0.8917602359128094 | 0.9721063100681742 | estimated |
| ... | 13 | 1800 | 2080 | 0.911154541638906 | 0.8675909974310361 | 0.9413541938308738 | 0.9570804788211932 | 0.9010584612453392 | 0.9820152111282763 | estimated |
| full_text_or_public_full_text_only | 1 | 98 | 98 |  |  |  |  |  |  | not estimated; fewer than two... |

## Deeks-type funnel asymmetry regression

| **k** | **slope** | **se** | **t** | **p_two_sided** |
| --- | --- | --- | --- | --- |
| 18 | 1.5753114530950825 | 13.29662093065055 | 0.1184745704424627 | 0.90716630601121 |

# Supplementary Table S8. Leave-One-Out Analysis

| **excluded_label** | **k** | **paired_exams** | **sensitivity** | **sens_ci_low** | **sens_ci_high** | **specificity** | **spec_ci_low** | **spec_ci_high** | **spec_i2_percent** |
| --- | --- | --- | --- | --- | --- | --- | --- | --- | --- |
| Motta 2012 | 17 | 2310 | 0.909371148775273 | 0.8706383903834709 | 0.937341383516095 | 0.938743392177908 | 0.8812007550098319 | 0.969382586823268 | 88.96423197178245 |
| Perini 2011 | 17 | 2057 | 0.9163196212661893 | 0.879521232059212 | 0.942611877095077 | 0.9469854229329475 | 0.8894388904862776 | 0.9754074363960192 | 88.38359240974565 |
| Johnsen 2020 | 17 | 2219 | 0.9159213907984466 | 0.880225008944274 | 0.9416840435912747 | 0.937302550450524 | 0.87884880390376 | 0.9685620611262864 | 88.31230719235815 |
| Park 2022 | 17 | 2342 | 0.9064566932323198 | 0.8680474084566614 | 0.9345289090895729 | 0.9506865633990891 | 0.8971798896524702 | 0.9770607683677996 | 89.34581517813388 |
| Gurtler 2013 | 17 | 2252 | 0.9000985983491104 | 0.8621943215211936 | 0.9284423638550655 | 0.9463224161805062 | 0.8904634587562567 | 0.9745110749477685 | 89.11874832990866 |
| Ten Bosch 2010 | 17 | 2325 | 0.9147926916566337 | 0.878446480485352 | 0.9410008052535048 | 0.9462379801060519 | 0.9104979685245304 | 0.9682046467881537 | 71.541498095193 |
| Houdek 2015 | 17 | 2424 | 0.9093488046422619 | 0.8716305926321747 | 0.936788165532793 | 0.9431572073639021 | 0.8882480775856487 | 0.9719391397010535 | 89.32306314125934 |
| Bredahl 2016 | 17 | 2174 | 0.9155067902918483 | 0.877512175010327 | 0.9424882416847243 | 0.9447794356908314 | 0.8881324859888207 | 0.9735946004731346 | 88.46427106957327 |
| Faccioli 2018 | 17 | 2315 | 0.8974630628858629 | 0.8601902952392118 | 0.925657833214618 | 0.9413240511681386 | 0.8852056542443937 | 0.9709099436187204 | 89.20449701630366 |
| Clevert 2008 | 17 | 2409 | 0.9076107950562899 | 0.8702388912085111 | 0.9350230374441383 | 0.9454840007616554 | 0.8910423952120058 | 0.9735314356954933 | 89.34275942837498 |
| Henao 2006 | 17 | 2432 | 0.9093724589668856 | 0.8720126592881947 | 0.9366194852950376 | 0.9498057037714395 | 0.8988869086506864 | 0.975773761136769 | 89.3513179351399 |
| Iezzi 2009 | 17 | 2368 | 0.9045974433945314 | 0.8671501796673585 | 0.9323131256349066 | 0.9503959603253818 | 0.8980680614936508 | 0.9765618330269461 | 89.34002969963166 |
| David 2016 | 17 | 2271 | 0.9043368993666516 | 0.8669460478829141 | 0.9320438100258491 | 0.9380523891192845 | 0.8801795893095017 | 0.9689586452474455 | 88.88602019465579 |
| Cantisani 2011 | 17 | 2344 | 0.9070376268535363 | 0.8692350545700018 | 0.9347324919112925 | 0.9392840484089223 | 0.8820144808995206 | 0.969709910254307 | 89.02138304677457 |
| Cai 2017 | 17 | 2422 | 0.9109867959253544 | 0.8731327620316953 | 0.9383437018391697 | 0.9503236512090775 | 0.9000522255979745 | 0.9759843850781875 | 89.30965481892743 |
| Ma 2023 | 17 | 2249 | 0.9149039489115168 | 0.8779409664373399 | 0.9414203491581528 | 0.936977387673799 | 0.8782943967238573 | 0.9683836614134065 | 88.61131824434277 |
| Wang 2024 | 17 | 2354 | 0.9089644136286399 | 0.870181831464977 | 0.9369997007839223 | 0.9406683022413289 | 0.8836965073094607 | 0.9706588237611364 | 89.06224105840126 |
| Nijhawan 2024 | 17 | 2417 | 0.9070376268535363 | 0.8692350545700018 | 0.9347324919112925 | 0.9437849118130154 | 0.8893321295994517 | 0.9722799820806115 | 89.35285613347025 |

# Supplementary Figure S1

**PRISMA-DTA public lock-date flow diagram.**


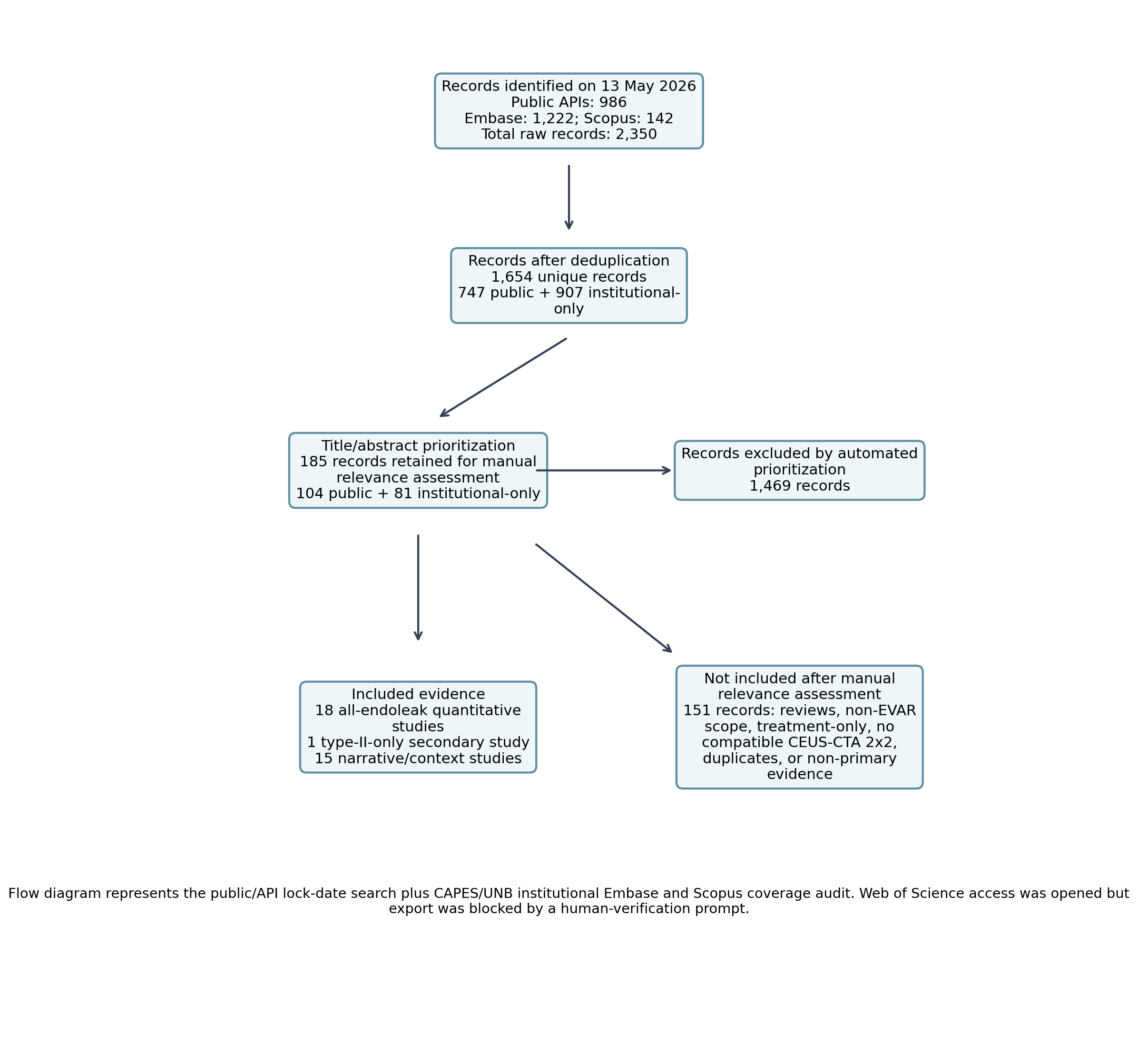

Supplement: Supplementary file 1 — Supplementary Material 1: Supplementary Methods S1. Search Strategy. Supplementary Methods S2. Lock-Date Search Summary. Supplementary Table S1. Study-Level Diagnostic Extraction. Supplementary Table S2. Primary-Pool 2 × 2 Diagnostic Tables. Supplementary Table S3. Reference-Standard and Unit-of-Analysis Audit. Supplementary Table S4. Final QUADAS-2 and QUADAS-C Mapping. Supplementary Table S5. Source Verification Matrix. Supplementary Table S6. Contemporary Narrative Evidence. Supplementary Table S7. Bivariate, Source-Basis, and Small-Study-Effect Analyses. Supplementary Table S8. Leave-One-Out Analysis. Supplementary Figure S1: PRISMA-DTA public lock-date flow diagram [file 42155_2026_731_MOESM1_ESM.docx]
